# Thermodynamic State Ensemble Models of *cis*-Regulation

**DOI:** 10.1371/journal.pcbi.1002407

**Published:** 2012-03-29

**Authors:** Marc S. Sherman, Barak A. Cohen

**Affiliations:** 1 Computational and Molecular Biophysics, Washington University in St. Louis, St. Louis, Missouri, United States of America; 2 Center for Genome Sciences, Department of Genetics, Washington University in St. Louis, St. Louis, Missouri, United States of America; Whitehead Institute, United States of America

## Abstract

A major goal in computational biology is to develop models that accurately predict a gene's expression from its surrounding regulatory DNA. Here we present one class of such models, thermodynamic state ensemble models. We describe the biochemical derivation of the thermodynamic framework in simple terms, and lay out the mathematical components that comprise each model. These components include (1) the possible states of a promoter, where a state is defined as a particular arrangement of transcription factors bound to a DNA promoter, (2) the binding constants that describe the affinity of the protein–protein and protein–DNA interactions that occur in each state, and (3) whether each state is capable of transcribing. Using these components, we demonstrate how to compute a *cis*-regulatory function that encodes the probability of a promoter being active. Our intention is to provide enough detail so that readers with little background in thermodynamics can compose their own *cis*-regulatory functions. To facilitate this goal, we also describe a matrix form of the model that can be easily coded in any programming language. This formalism has great flexibility, which we show by illustrating how phenomena such as competition between transcription factors and cooperativity are readily incorporated into these models. Using this framework, we also demonstrate that Michaelis-like functions, another class of *cis*-regulatory models, are a subset of the thermodynamic framework with specific assumptions. By recasting Michaelis-like functions as thermodynamic functions, we emphasize the relationship between these models and delineate the specific circumstances representable by each approach. Application of thermodynamic state ensemble models is likely to be an important tool in unraveling the physical basis of combinatorial *cis*-regulation and in generating formalisms that accurately predict gene expression from DNA sequence.

## Introduction

Modern molecular biology and genomics methods allow investigators to readily assay protein and mRNA expression levels and identify interactions between proteins, RNA, and other cellular components. Leveraging these data to understand the functional significance of interactions on gene expression is a key challenge in computational biology. The recent application of thermodynamic models to gene regulation is an exciting development, as each model reflects a specific, testable hypothesis regarding the physical architecture of the underlying molecular system [1]–[4]. Such models will help transform parts lists, which describe the components of regulatory systems, into models that integrate the interactions between components into accurate predictions of gene expression.

Though a gene is regulated at every step of transcription and translation, a large component of regulation operates at the level of the promoter [5]. Transcription factors bind to specific sequences and modulate transcription by influencing exposure of the polymerase binding site (chromatin remodelers [6]), chemically modifying DNA (methyltransferases [7]), and recruiting factors necessary for, or inhibitory of, polymerase complex formation [8]–[11]. These mechanisms constitute the *cis*-regulatory component of a gene's regulation. Understanding gene expression under a variety of cellular contexts requires a well-grounded theory for modeling *cis*-regulatory function.

Here we show the biochemical derivation of the thermodynamic framework used to model promoter activity. The derivation is presented in a form that can be readily coded in any programming language, allowing readers to develop *cis*-regulatory models specific to their own systems. We suggest how this approach can be leveraged to model virtually any *cis*-regulatory mechanism. We also demonstrate that modular Michaelis-like functions, another commonly used framework, are a specific subset of the thermodynamic model framework. To demonstrate this, we recast Michaelis-like functions as thermodynamic models, highlighting the physical assumptions necessary for interconversion. Viewing Michaelis functions in this form reinforces the principles of the thermodynamic framework, emphasizes the relationship between these approaches, and provides criteria for an investigator to choose an appropriate *cis*-regulatory model. The flexibility of the thermodynamic framework, along with its grounding in basic physical principles, makes it a powerful tool for unraveling the molecular interactions that underlie combinatorial *cis*-regulation.

## *cis*-Regulatory Functions in Models of Transcription

A model of *cis*-regulation relates the activities of various transcription factors acting on gene 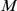 to the concentration of mRNA produced by transcription of 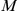. To illustrate how *cis*-regulation contributes to expression, a general model of transcription, derived from physicochemical principles, is presented [12]. The concentration of any particular mRNA species, denoted as 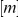, changes over time according to the first order rate equation (Equation 1),
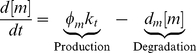
(1)where 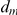 is the degradation rate constant of the RNA transcript in units of inverse time, 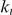 is the concentration of RNA transcripts generated per unit time when RNA polymerase is committed to transcription, and 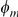 is the probability that a DNA template is committed to transcription. The quantity 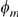 is the *cis*-regulatory term. The 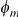 function integrates elements of the cellular milieu that affect transcription and outputs the probability that a single DNA molecule is committed to transcription. In a clonal population of cells at equilibrium, 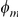 equals the fraction of those cells currently committed to transcription. Although there are numerous other discrete, continuous, and stochastic models of gene expression [12], [13], every model must contain some form of the *cis*-regulatory function 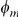.

## Anatomy of a *cis*-Regulatory Function

Importantly, there is no hypothesis-independent form of the *cis*-regulatory function; *any* choice of 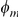 is a hypothesis about the mechanism of gene M's transcription. Even if we choose 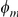 to be a constant, we imply that gene M is constitutively transcribed at a rate unaffected by any cellular or environmental factors. There is no single correct formulation of 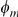; investigators must formulate 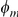 based on aspects of their system they know to be true, and on hypotheses they hold regarding the important features of their system.

Two approaches have been used to formulate *cis*-regulatory 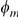 expressions: (1) Michaelis-like functions and (2) thermodynamic state ensemble models. Michaelis-like functions have been most frequently employed to study large gene regulatory networks [12], [14]–[18], owing to their modular design and limited number of free parameters. State ensemble approaches have been the model of choice for characterizing a few specific genes in great detail [1]–[3], [19]–[23]. By manipulating these two approaches analytically, we will show that the Michaelis-like models are a specific case in the thermodynamic framework, thus uniting these two approaches and also illuminating some of the subtleties of the Michaelis-like models.

### Thermodynamic State Ensemble Approach

The “thermodynamic model” is a framework for constructing a set of states that collectively encode the rules of transcription for a particular promoter. Each state represents a particular number and arrangement of transcription factors bound to a DNA template. Some states are transcriptionally active while others remain transcriptionally dormant. All states occur at some point, but their contributions to transcription are weighted by their relative stabilities. In this formulation, 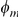 is the probability of a promoter being in a transcriptionally active state. The essence of the thermodynamic framework is to compute the ratio of transcriptionally active promoter states to the sum of all states, active and inert. This ratio depends on variables including the exact *cis*-regulatory sequences present in the promoter, the concentrations of proteins that bind these sequences, and the affinities of the protein–DNA and protein–protein interactions that occur on the DNA. The thermodynamic formalism provides a flexible framework in which to account for molecular interactions that control *cis*-regulation.

**Figure 1:**
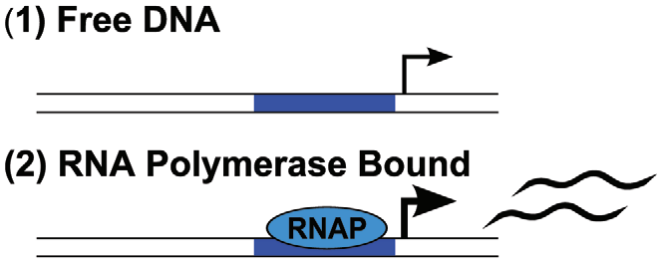
States of a basal promoter. A basal promoter is composed of two states, one where DNA is bound with RNAP and is transcriptionally active, and another where DNA is free and inactive.

Generating a model requires writing down all possible states a promoter may adopt in the form of a binding polynomial, 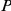 [2], [24]. To illustrate the binding polynomial, we first consider the simple case of a basal promoter (Figure 1). Defining what is meant by basal transcription is central to the development of a model framework because activation and repression reflect changes relative to the basal level of transcription. Here, a basal promoter is a DNA template that contains a binding site for RNA polymerase (RNAP) and no other *cis*-regulatory sequences. Basal transcription is defined as the level of transcript produced by RNAP in the absence of regulation by transcription factors. Note that RNAP serves as a proxy for the rate limiting step of transcription, whether that be the recruitment of a particular co-factor to the RNAP holoenzyme, or binding of a specific transcription factor. The binding polynomial for the basal promoter is given in Equation 2. For reference, Box 1 contains definitions relevant for the derivation.
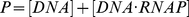
(2)

**Box 1. Definitions**

**Basal promoter:** a promoter in which the sequence codes only for binding of RNA Polymerase.

**Basal transcription:** the RNA expression level attained by driving a gene with a basal promoter.

**Binding polynomial:** a mathematical expression calculated by summing the concencentrations of all states of a particular macromolecule (in this case, DNA).

***cis*** **-regulatory site:** a specific sequence recognized and capable of being bound by a transcription factor or polymerase.

**Cooperativity:** a binding modality in which the occupancy of a state where two or more factors are bound to DNA is not equal to the occupancy expected if each factor were to bind independently. In terms of energy, which is additive: if factors A and B bind independently, then the energy of the state where both are bound is 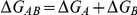. If the actual energy, 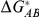, is not equal to 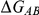, then there exists some interaction between A and B such that 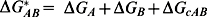, where 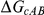 is the cooperative energy term of this interaction. 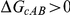 reflects positive cooperativity, or an adhesive interaction between A and B, while 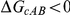 indicates negative cooperativity, or a repulsive interaction between A and B.

**Equilibrium:** when either the time average or population average of all relevant concentrations of biochemical species are not changing.

**Equilibrium binding constant:** in an interaction between biochemical species, the equilibrium binding constant is the equilibrium concentration of the product divided by the product of the equilibrium concentrations of all reactants. In a cellular equilibrium, as defined above, these are actually “apparent” equilibrium constants.

**Macroscopic binding constant:** an analog to the total energy required to bind all species in a state from an unbound state. If only two species are interacting, the macroscopic binding constant equals the equilibrium binding constant. In all other situations, the macroscopic binding constant is equal to the product of all equilbrium binding constants neccesary to convert two or more species from the free to bound state.

**Partition function:** the binding polynomial normalized by the concentration of a reference state (in this case, free DNA, [DNA]). The probability of observing a particular state may be calculated by dividing a state contained in the partition function by the total partition function.

**Promoter:** the sequence adjacent to the coding region of a gene containing RNA polymerase binding sequence and any other *cis*-regulatory binding sequences.

**RNA polymerase (RNAP):** the biochemical machinery needed for basal expression. In the context of an experiment it can also be thought of as the aspect of the experiment not being altered.

**State:** a specific arrangement of transcription factors and/or RNAP bound to DNA.

**Transcription factor (TF):** any protein capable of both binding a promoter and affecting expression by influencing the polymerase's ability to bind DNA and/or transcribe.

This DNA-centric binding polynomial enumerates the two mutually exclusive states of a basal promoter; either DNA is free or bound by RNAP. From *P*, we can determine the fraction of DNA bound with RNAP, 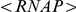. At equilibrium, this is the concentration of bound DNA divided by the total concentration of DNA, 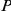 (Equation 3).
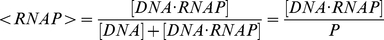
(3)


Equation 3 is a basic *cis*-regulatory function (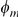) for a basal promoter where concentration of bound polymerase is the only determinant of transcription. The primary assumption of the thermodynamic model, originally introduced by Shea and Ackers [2], is that binding of the polymerase complex is the key event leading to production of a transcript, and that other proteins affecting expression operate by recruiting or inhibiting the polymerase complex. Thus, the fraction of polymerase complex bound is directly proportional to the number of transcripts produced.

We can reformulate Equation 3 in terms of its component free species and their association constants. The apparent association constant for the binding of RNAP to DNA is 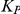.
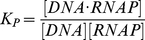
(4)


This simplification presumes that the concentrations of all cofactors required to form the RNAP complex are invariant. Solving for 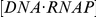 in Equation 4 and substituting it into Equation 3 results in Equation 5. The [DNA] factor is present in all terms and is subsequently dropped.
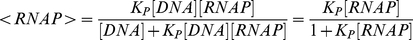
(5)


The denominator of the right-hand-side of Equation 5 is called the biochemical partition function (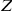) for our system, and is exactly equal to 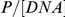. Dividing any state or sum of states 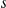 listed in 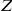 by 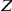 results in the probability of observing 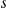. The reference state, where DNA is unbound, is represented by the 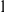 in 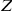; consequently, the probability of finding DNA unbound is 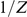. Equation 5 is perhaps the most intuitive form of the thermodynamic model as it shows clearly the origin of each state. Each state is a summand, and the elements within a summand serve as a sort of recipe for how to make that state. For example, the numerator term in Equation 5 can be read as “binding of RNAP to DNA has an equilibrium binding constant of 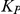
”. This form is particularly useful because it expresses the model in terms that are more accessible to experiment. While *in vivo* binding constants and concentrations of free species are difficult to determine, reasonable proxies for these quantities can often be obtained experimentally [20], [25].

Several other manipulations of these equations are employed in the literature. In addition to writing states in terms of free species concentrations, Shea and Ackers substitute association constants with Boltzmann weights [2]. Others course-grain the product of association constants and concentrations into single parameters [1], [20], reducing computational complexity. These manipulations to the free species form described above are discussed in the supplement (Text S1, Alternate *cis*-Regulatory Function Forms).

### Building a Thermodynamic State Ensemble Model

The framework suggested by Shea and Ackers allows great flexibility for assembling models to reflect a wide variety of mechanisms and behavior.

**Figure 2:**
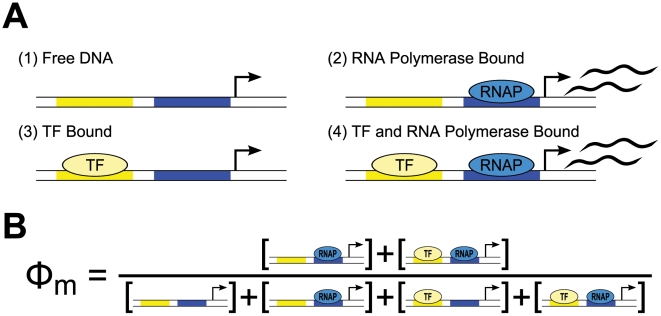
Thermodynamic state ensemble model example. (A) Four states are allowed in this example, two where transcription is inactive (states 1 and 3) and two states where transcription is active (states 2 and 4). (B) The 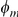 function is composed of the concentrations of transcriptionally active states summed in the numerator divided by the sum of the concentrations of all possible states.

For any particular system, construction of a thermodynamic *cis*-regulatory function requires three components: (1) a list of all states, (2) the macroscopic equilibrium constant for each state, and (3) a boolean for whether each state is capable of transcribing or not. We will illustrate the formalism using a promoter with a single binding site for a transcription factor and a binding site for RNA polymerase (Figure 2). We have introduced linear algebra to showcase the building blocks of the framework while demonstrating how to code a specific model. We will encode the list of states in a position matrix ***L***, which we will then convert into the functional state vector ***s***.
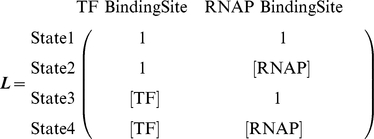
States are written as a function of position with concentrations representing what can bind each position in each state. A “1” denotes nothing is bound in that particular position and state. The product of all terms in each state are used to generate the state vector 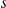. Unique states in 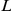 may result in degenerate states in 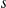. This example with two sites requires two columns; other, more complicated systems with multiple sites are modeled by adding new columns.
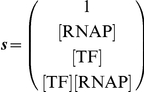
State 1, the first row of ***s***, corresponds to the reference state where DNA has nothing bound. State 2 has RNAP bound by itself, state 3 has TF bound by itself, and state 4 has both TF and RNAP bound. Simply by writing these states we are already specifying the architecture of our system. For example, if RNAP were to require TF to be present before it binds, then state 2, where RNAP is bound by itself, would not exist and would not be included among the list of possible states.

**Figure 3:**
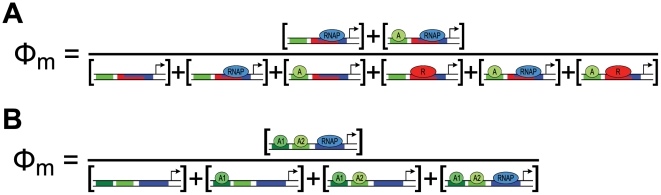
Graphical representations of thermodynamic *cis*-regulatory functions. Proteins/complexes are represented as ovals, binding sites as rectangles. (A) Repressor-RNAP competition with activator release model, see Box 2. The ovals represent RNAP (blue), repressor (red), and activator (green). Note that the repressor and RNAP binding sites are overlapping to reflect competition between sites. (B) Sequential binding model, see Box 2. The ovals correspond to RNAP (blue), activator A1 (dark green), and activator A2 (light green).

Vector ***b*** contains the macroscopic equilibrium constants 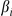 for each state 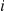; as such, it will be the same length as ***s***. Macroscopic equilibrium constants reflect the energy difference between that state and the reference (unbound) state, and comprise the product of the stepwise equilibrium constants in the intervening steps.
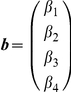
The macroscopic binding constant for the reference state is always 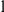, representing free [DNA]; thus, 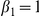 (see Equation 5).

Lastly, we define vector ***t***, which contains boolean values for whether a state is capable of transcribing. For example, we might assume that transcription occurs any time RNAP is bound, as assumed by Shea et al. [2]. Changes in the values of the ***t*** vector can accommodate situations where this assumption proves to be false.
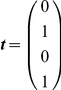
The *cis*-regulatory function 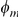 is the sum of states capable of transcribing divided by the sum of all possible states. The denominator of 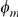 is the biochemical partition function 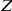, which can be expressed as the dot product of the transpose of ***s*** with ***b***. Taking the pairwise element product of ***b*** and ***t*** results in vector 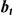.
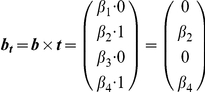
Then the dot product of the transpose of ***s*** with 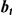 yields the sum of transcriptionally active states.

Generally, for any architecture ***L*** written as a vector ***s*** that contains the concentrations of all relevant species, vector ***b*** containing the macroscopic equilibrium constants for each state, and vector ***t*** relating whether a state is capable of transcribing, the *cis*-regulatory function is:
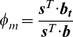
(6)


For our example,
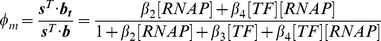
(7)


The 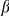 terms reflect the most general treatment of this system, but can also be written as functions of their stepwise equilibrium constants. In the scenario above, the macroscopic equilibrium constant 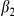 is exactly equal to the equilibrium constant for binding of RNAP to DNA, denoted as 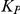. Similarly, 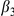 is exactly equal to the equilibrium constant for association of TF to DNA, denoted as 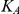. 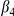 can be a number of different expressions depending on the system. For example, setting 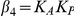 implies completely independent non-cooperative binding of TF and polymerase; that is, binding of one does not influence binding of the other. In this case,
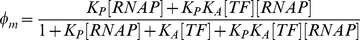
(8)


Completely independent binding of transcription factor and RNAP implies that the presence of TF has no bearing on the probability of RNAP being bound, a scenario reflected in the equation by factoring and canceling out the TF terms, revealing our basal promoter function:
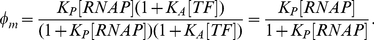
(9)


In order for the TF to affect binding of the polymerase we must introduce a cooperative binding term 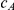. Then 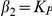 and 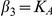 as before, but 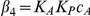. The new 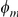 no longer simplifies to the trivial case.
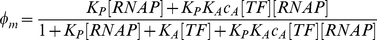
(10)


The cooperative term 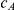 reflects the energy associated with the interaction of the polymerase with the TF. If 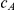 = 1, we recover the case above where binding of the TF has no bearing on the binding of the polymerase. If 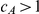, the TF acts like an activator; if the TF is bound, it stabilizes the state where polymerase is also bound. Conversely, if 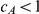, the TF acts like a repressor; TF binding decreases the stability of the state where polymerase is also bound. See Box 2 for additional examples.

**Box 2. Example Model Implementations**

**Repressor-RNAP competition with activator release model.** In this example, RNAP is blocked from binding by a repressor, R, bound to the same site. Activator A binds to an adjacent site and, through negative cooperativity, ejects the repressor from DNA, thus freeing the RNAP binding site. Neither activator nor repressor interacts directly with polymerase. The ***b*** vector element 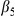 contains the cooperative term 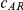, which needs to be less than one for the activator and repressor to repel each other. Note that the repressor and polymerase need not have exactly the same binding site, as long as the presence of one excludes binding of the other (see Figure 3A). This general principle of allowing or disallowing states can be expanded to account for promoters with overlapping binding sites [2], [30].
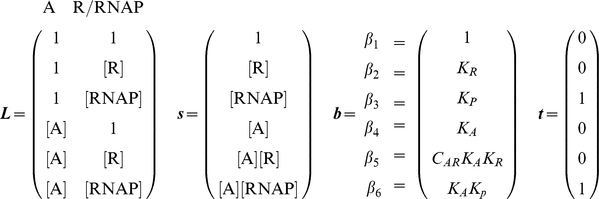


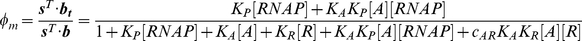

**Sequential binding model.** Several known regulatory mechanisms involve sequential binding of activators. In this model, activator A1 permits binding of activator A2, which in turn recruits polymerase. This model subscribes to Michaelis-like model logic where all activators are required for binding, but the sequential aspect can only be captured using a state ensemble approach. We have engineered sequential binding by disallowing activator A2 to bind without activator A1, and disallowing polymerase to bind without activator A2 (see Figure 3B).
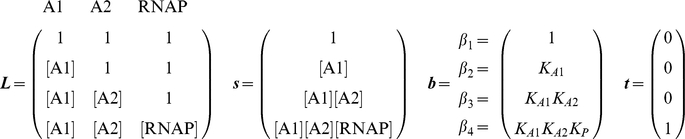


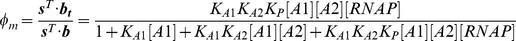

When constructing a thermodynamic model, an investigator explicitly selects the number of binding sites, decides which proteins bind to each site, determines whether a state is transcriptionally active, and assigns cooperative interactions between binding partners. The resulting *cis*-regulatory function's numerator contains transcriptionally active states while the denominator encodes all binding states. These traits confer considerable versatility to the thermodynamic modeling approach, making it a powerful tool for exploring *cis*-regulatory control of gene expression.

### Modular Michaelis Functions

Modular Michaelis-like functions have also been used to model *cis*-regulation. Ronen et al. introduced activator and repressor equations (Equations 11 and 12) as Michaelis-Menten kinetic equations to model transcription temporally [26]. Various groups [12], [14]–[18] subsequently used these equations as *cis*-regulatory input functions because increases in activator concentration ([A]) or activator efficiency (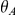) monotonically heighten expression (Equation 11), while increases in repressor concentration ([R]) or efficiency (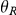) monotonically diminish expression (Equation 12). However, these equations are not derived from the classical Michaelis-Menten enzyme-substrate system and bear no relation other than mathematical form, hence our use of the term “Michaelis-like.” 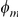, the *cis*-regulatory function, is formulated as the product of 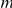 activator (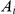) and 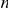 repressor (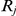) functions (Equation 13),
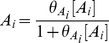
(11)

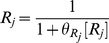
(12)

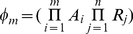
(13)where 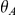 and 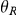 were originally defined as apparent affinities of activator and repressor for their promoter sites [14], [26], but later interpreted as efficiencies of activation or repression [15]. Importantly, this Michaelis-like formulation necessarily results in an AND-type circuit where expression occurs only if ALL activator factors are bound AND ALL repressors are NOT bound to DNA [12].

One subtlety of the Michaelis-like models is that there is no uniform definition of the basal rate. To illustrate this, consider two promoters, one with a single binding site for an activator and the other with a single site for a repressor. The corresponding models are given in Equations 14 and 15:
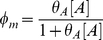
(14)

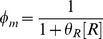
(15)


One might expect that removing the effect of the TF in either the single activator or single repressor model would cause reversion to the same basal rate. This is not the case. In the single activator model setting, 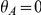 or 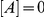 results in a basal rate of zero, the minimum possible. In contrast, setting 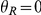 or 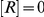 in the single repressor model results in a basal rate of 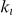, the maximum possible. Investigators must be aware of the context-dependent definition of the basal rate when formulating appropriate Michaelis-like models of their systems.

## Modular Michaelis Functions as Partition Functions

What is the physical interpretation of the Michaelis function architecture? By converting the Michaelis model formulations above (Equation 13) into thermodynamic functions we will reveal assumptions underlying Michaelis-like models that are not obvious in their original formulation. The steps involved in converting one model to the other also highlight the similarity between these models, and demonstrate that the Michaelis formulation is simply a thermodynamic model with specific *cis*-regulatory rules.

We can reconcile the thermodynamic model with the Michaelis framework by treating polymerase as an activator. Since polymerase is required for transcription, we incorporate the basal thermodynamic function (Equation 5) into the Michaelis-like formulation, Equation 13, as an activator function (Equations 16 and 17).
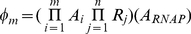
(16)

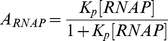
(17)


Comparing Equations 16 and 17 with Equation 13 illustrates that the original Michaelis-like function requires the assumption that 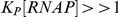, such that the activator function for polymerase 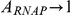. In other words, the Michaelis approach assumes that the polymerase site is saturated, or always occupied.

The asymmetry in the way Michaelis functions treat RNAP becomes clear when they are recast in the thermodynamic framework. Consider the following Michaelis-like models: activator only (Equation 18), repressor only (Equation 19), and one activator and one repressor (Equation 20).
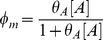
(18)

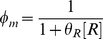
(19)

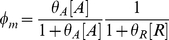
(20)


Adding in the polymerase function as in Equation 16 and multiplying out the terms, we generate the following expressions.
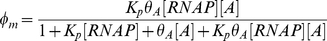
(21)

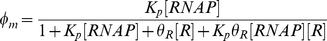
(22)

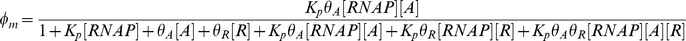
(23)


Comparing the resulting models shows that the Michaelis-like activator and repressor functions treat the state in which only RNAP is bound very differently. A one activator promoter (Equation 21) transcribes only when both RNAP and activator are present, as represented by the sole numerator term. The presence of the 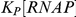 term indicates that polymerase *can* bind DNA without activator, but because this state is only in the denominator, binding does not result in transcription. In contrast, the repressor model (Equation 22) only transcribes when RNAP is bound and repressor R is *not* bound, as reflected by the 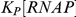 state being the sole numerator term. Thus, the presence of repressor inhibits expression absolutely. In order to appropriately model their own systems with Michaelis-like functions, investigators should be aware of the different interpretation of the RNAP-only state in the activator and repressor functions.

Recasting the original Michaelis-like functions as a thermodynamic ensemble model also highlights its implicit AND-circuitry. The inclusion of both an activator and repressor in the Michaelis-like formulation results in a model with only a single term in the numerator (Equation 23). This means that transcripts are generated only when activator is bound and repressor is not bound. Higher numbers of transcription factors continue these patterns. For example, a two or more activator model requires that all activators are bound for transcription, and a two or more repressor model requires that none of the repressors are bound. In a mixed system with multiple activators and repressors, the trend set by the one activator and one repressor model (Equation 23) prevails; transcripts are produced only when all activators accompany polymerase with no repressors present. Investigators must decide on the validity of this constraint when employing Michaelis-like functions.

The implicit AND logic associated with Michaelis-like functions leads to a seeming paradox. The more activators a promoter contains, the lower its expression. This is because the probability of having all activators bound at the same time decreases with the number of activator binding sites in a promoter. This seeming paradox and the general AND-circuitry associated with this formalism led some groups to produce an OR-logic function for activators (Equation 24) and repressors (Equation 25) [16], [17]:
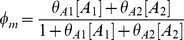
(24)

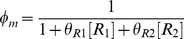
(25)


The activator function involves addition rather than multiplication of individual transcription factor effects. Following the same steps outlined above, one can show that the OR-logic model here no longer produces zero expression when any single activator concentration (or affinity) goes to zero. However, if all activator concentrations are zero, transcription is abolished, implying that some activator (of either type) is required to produce transcripts.

To allow basal expression even in the absence of transcription factors, some groups [14], [16], [17] introduce an empirical basal leak term to the Michaelis function formulation. Leak functions can also be reformulated as thermodynamic models, revealing a similar set of implicit physical assumptions (see Text S1, Michaelis-like Functions with Basal Leak).

These are reasonable models provided that the mechanisms described appropriately reflect the logic of the system being modeled. Michaelis-like functions can be a simple and powerful framework for modeling many types of regulatory logic. The purpose of reformulating these models in the thermodynamic framework was to demonstrate that Michaelis-like functions are simply one type of thermodynamic model. The assumptions that underlie these particular models, which are easy to see in the thermodynamic framework, are likely to be valid for many, but not all types of *cis*-regulatory logic.

Some regulatory mechanisms require the use of the more general thermodynamic framework. For example, a repressor might function by directly blocking polymerase binding, so that simultaneous binding of polymerase and repressor does not occur [2]. Or, an activator might boost expression, but transcription continues even in the absence of activator [5]. Michaelis-like functions can be applied in these situations, but cannot distinguish between various mechanisms. Box 2 illustrates two examples of *cis*-regulatory architectures that can only be represented using the more general thermodynamic approach.

## Hill Cooperativity in the Context of a *cis*-Regulatory Function

Cooperativity is a repulsion or attraction between proteins on the surface of DNA such that the sum of the free energies of proteins binding independently differs from the energy of the proteins binding together. We discussed cooperativity in the thermodynamic framework using Equation 10. Another commonly used method to capture cooperativity is the addition of Hill coefficients (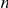) to the Michaelis-like functions [14], [16], [18], [27]. For example, the modified one activator and one repressor models correspond to:
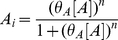
(26)

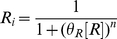
(27)


These functions are known as Hill functions [24]. Hill functions measure cooperativity by quantifying deviation from independent binding in a traditional biochemical binding titration experiment. Used as *cis*-regulatory functions, Goutsias and Kim point out that these functions imply extreme cooperativity; for two proteins, *n* = 2 implies that the proteins can only bind simultaneously, never independently [12]. We will show the origin of this assumption again using the thermodynamic formalism.

### Extreme Cooperativity

The assumption of extreme cooperativity must be made in order to convert the thermodynamic model into a Hill function. Consider a promoter with two binding sites for an activator, A. The two A proteins exhibit positive cooperative binding with constant 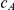 (where 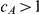). Because we want to compare our model directly to the Hill-like model, we make the Michaelis assumption that both activators must be present for transcription to occur. Following the steps leading up to Equation 7, we produce the following *cis*-regulatory function:
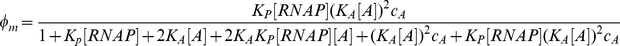
(28)


This model is not directly comparable to the Hill function in Equation 26. In order to reduce this model to a form that is comparable to the Hill model, we must further assume that the TF affinity for DNA is small and the cooperative binding constant large (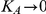, 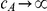). Under this assumption all terms containing 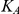 without an accompanying 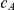 disappear:
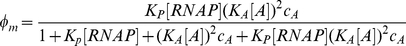
(29)


The polymerase binding term can now be factored out.
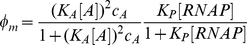
(30)


The right hand term in Equation 30 is the basal promoter function and the left hand term is the new activator function, which is now directly comparable to Equation 26. The key point is that in order to convert the thermodynamic framework into the Hill framework we must assume that 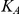 is tiny and that 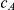 is large. The physical interpretation of this assumption is that the transcription factors can only bind together, never independently. This comparison reveals other subtleties regarding Hill function–based cooperativity. Comparing Equation 26 with the left-hand term in Equation 30, and setting the Hill coefficient 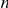 equal to 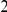, we find that 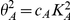. This provides some physical intuition into the meaning of the theta term in the Michaelis-like framework. In addition, we again have to assume that polymerase is in excess so that the right-hand term of Equation 30 goes to one. In summary, a Hill coefficient of 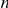 corresponds to 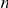 identical transcription factors binding with extreme cooperativity (either none or 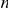 are bound at a given time) to a promoter with 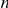 TF binding sites. Like the Michaelis formalism, all activator TFs must be bound to initiate expression. This exercise also demonstrates that non-integer values of 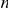 correspond to fractions of proteins binding DNA, and should thus be used with caution [12], [14].

A practical realization of extreme cooperativity is the oligomerization of TFs prior to binding. While the model above implies that TFs are monomeric in solution and 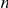-mers only at the promoter, it is relatively simple to include 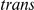 binding events into the system. In the supplement (Text S1, Oligomerization with Hill Functions) we show how 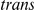 oligomerization binding constants contribute to the 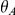 parameter of the Hill equation.

## Discussion

Using expression-profiling methods, investigators routinely collect large quantities of gene expression data. A mature and robust quantitative framework would draw meaningful conclusions from these rich but complex datasets. Here we derived a thermodynamic state ensemble framework for capturing *cis*-regulatory architectures. Our intention here was to clarify the assumptions of the thermodynamic framework, to provide a step-by-step guide for constructing such a model, and to impart guidance in interpreting the physical meaning of the parameters of these models. Different investigators will collect different types and amounts of data, in turn requiring pre- and post-processing steps specific to their respective systems. This includes data filtering and fitting routines for parameter estimation that we could not address here and must be dealt with on a case-by-case basis. What we did attempt to address were the aspects of thermodynamic modeling that will be common to all investigators; namely the construction and interpretation of such models.

The flexibility of the thermodynamic formalism makes it simple to model different promoter architectures and regulatory mechanisms. Discrete promoter states determine the overall architecture of the model, with individual states constructed from the product of activities of DNA-bound molecules. The balance between productive and silent states determines the probability of transcription (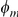), a term mathematically composed of a denominator comprising the sum of all states and a numerator containing the sum of transcriptionally active states. Selecting whether a state is transcriptionally active, and even whether a state exists at all, allows a large number of possible models to be constructed. With this versatility comes both a warning and a virtue; any architecture devised reflects a specific hypothesis about the physical system being modeled.

Michaelis-like models are simplified forms of the thermodynamic framework. Each type of Michaelis-like *cis*-regulatory function can be derived from the thermodynamic model framework by making a few key assumptions. Understanding these assumptions will help investigators to choose appropriate models for their systems. Michaelis models generally assume that polymerase is present in excess and that each transcription factor included acts at an independent site. Products of Michaelis-like functions represent the hypothesis that all activators, and no repressors, must be bound to initiate transcription. Sums of Michaelis functions correspond to situations in which at least one activator must be bound for transcription to occur (basal transcription is disallowed). A thermodynamic reformulation of Hill functions reflect a specific type of cooperativity in which either a site is free, or bound by 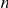 proteins, for a Hill coefficient of 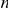. Alternatively, a Hill coefficient of 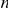 can imply binding of an 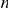-mer to the promoter. These two situations imply two distinct interpretations for the 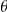 parameters. Michaelis and Hill-like functions are valid simplifications of the thermodynamic framework. It is up to individual investigators to decide when the assumptions underlying these simplifications are appropriate.

In some cases, investigators must employ the more general form of the thermodynamic framework. For example, repressors might inhibit transcription by binding directly to the RNAP binding site, a mode of repression that cannot be specifically represented using the Michaelis formulation. Such a mechanism can be captured by a thermodynamic state ensemble model in which one disallows the state in which both RNAP and repressor are simultaneously bound (for examples, see Box 2). In general, it may be wise to first cast any system under study in the thermodynamic framework before simplifying to the corresponding Michaelis model so that the underlying assumptions about the system are clear.

With the exception of a few well-characterized systems like *lac* and the 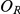 lysis-lysogeny operator of 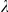-bacteriophage, combinatorial *cis*-regulation of genes is not understood to the point where one can predict levels of transcription from the *cis*-regulatory content of a gene. The parts list of *cis*-regulatory components is growing rapidly; soon we will know the binding preferences of all transcription factors and their activating or repressing activities [28], [29]. Even with this catalog in hand, we will not understand gene regulation until we understand how the interactions between *cis*-regulatory components generate specific patterns of transcription. We are optimistic that the thoughtful application of state ensemble models will provide mechanistic insight into the physical interactions that underlie combinatorial *cis*-regulation.

## Supporting Information

Text S1 **Supporting Information.** Text S1 provides additional detail about alternative forms of the *cis*-regulatory expressions, discusses Michaelis-like functions in which there is a leak term and how these are related to the thermodynamic model framework, and demonstrates how *trans* binding events can be incorporated into a *cis*-regulatory function.
(PDF)

## References

[1] Buchler NE, Gerland U, Hwa T (2003). On schemes of combinatorial transcription logic.. Proc Natl Acad Sci USA.

[2] Shea MA, Ackers GK (1985). The or control system of bacteriophage lambda. a physical-chemical model for gene regulation.. J Mol Biol.

[3] Segal E, Raveh-Sadka T, Schroeder M, Unnerstall U, Gaul U (2008). Predicting expression patterns from regulatory sequence in drosophila segmentation.. Nature.

[4] Bintu L, Buchler NE, Garcia HG, Gerland U, Hwa T (2005). Transcriptional regulation by the numbers: models.. Curr Opin Genet Dev.

[5] Ptashne M, Gann A (2002). Genes and signals.

[6] Luo RX, Dean DC (1999). Chromatin remodeling and transcriptional regulation.. J Natl Cancer Inst.

[7] Klose RJ, Bird AP (2006). Genomic dna methylation: the mark and its mediators.. Trends Biochem Sci.

[8] Gruber TM, Gross CA (2003). Multiple sigma subunits and the partitioning of bacterial transcription space.. Annu Rev Microbiol.

[9] Métivier R, Penot G, Hübner MR, Reid G, Brand H (2003). Estrogen receptor-alpha directs ordered, cyclical, and combinatorial recruitment of cofactors on a natural target promoter.. Cell.

[10] Gorski SA, Snyder SK, John S, Grummt I, Misteli T (2008). Modulation of rna polymerase assembly dynamics in transcriptional regulation.. Mol Cell.

[11] van Essen D, Engist B, Natoli G, Saccani S (2009). Two modes of transcriptional activation at native promoters by nf-kappab p65.. PLoS Biol.

[12] Goutsias J, Kim S (2004). A nonlinear discrete dynamical model for transcriptional regulation: construction and properties.. Biophysical journal.

[13] Goutsias J, Lee NH (2007). Computational and experimental approaches for modeling gene regulatory networks.. Curr Pharm Des.

[14] Sneppen K, Krishna S, Semsey S (2010). Simplified models of biological networks.. Annu Rev Biophys.

[15] Kuttykrishnan S, Sabina J, Langton LL, Johnston M, Brent MR (2010). A quantitative model of glucose signaling in yeast reveals an incoherent feed forward loop leading to a specific, transient pulse of transcription.. Proc Natl Acad Sci U S A.

[16] Alon U (2007). An introduction to systems biology: design principles of biological circuits.

[17] Mangan S, Alon U (2003). Structure and function of the feed-forward loop network motif.. Proc Natl Acad Sci U S A.

[18] Monk NAM (2003). Oscillatory expression of hes1, p53, and nf-kappab driven by transcriptional time delays.. Curr Biol.

[19] Gertz J, Gerke JP, Cohen BA (2010). Epistasis in a quantitative trait captured by a molecular model of transcription factor interactions.. Theor Popul Biol.

[20] Gertz J, Siggia ED, Cohen BA (2009). Analysis of combinatorial cis-regulation in synthetic and genomic promoters.. Nature.

[21] Bintu L, Buchler NE, Garcia HG, Gerland U, Hwa T (2005). Transcriptional regulation by the numbers: applications.. Curr Opin Genet Dev.

[22] Fakhouri WD, Ay A, Sayal R, Dresch J, Dayringer E (2010). Deciphering a transcriptional regulatory code: modeling short-range repression in the drosophila embryo.. Mol Syst Biol.

[23] Zinzen RP, Senger K, Levine M, Papatsenko D (2006). Computational models for neurogenic gene expression in the drosophila embryo.. Curr Biol.

[24] Wyman J, Gill SJ (1990). Binding and linkage: functional chemistry of biological macromolecules.

[25] Mukherjee S, Berger MF, Jona G, Wang XS, Muzzey D (2004). Rapid analysis of the dnabinding specificities of transcription factors with dna microarrays.. Nat Genet.

[26] Ronen M, Rosenberg R, Shraiman BI, Alon U (2002). Assigning numbers to the arrows: parameterizing a gene regulation network by using accurate expression kinetics.. Proc Natl Acad Sci USA.

[27] Rosenfeld N, Elowitz MB, Alon U (2002). Negative autoregulation speeds the response times of transcription networks.. J Mol Biol.

[28] Berger MF, Bulyk ML (2009). Universal protein-binding microarrays for the comprehensive characterization of the dna-binding specificities of transcription factors.. Nat Protoc.

[29] Consortium EP, Birney E, Stamatoyannopoulos JA, Dutta A, Guigó R (2007). Identification and analysis of functional elements in 1genome by the encode pilot project.. Nature.

[30] Foat BC, Morozov AV, Bussemaker HJ (2006). Statistical mechanical modeling of genome-wide transcription factor occupancy data by matrixreduce.. Bioinformatics.

