# Supplementary material for: Thermodynamic State Ensemble Models of cis-Regulation
Source: PLoS Comput Biol. 2012 Mar 29;8(3):e1002407. doi: 10.1371/journal.pcbi.1002407 (PMC3315449; doi:10.1371/journal.pcbi.1002407)
Supplement: Text S1 — Supporting Information. Text S1 provides additional detail about alternative forms of the cis-regulatory expressions, discusses Michaelis-like functions in which there is a leak term and how these are related to the thermodynamic model framework, and demonstrates how trans binding events can be incorporated into a cis-regulatory function. (PDF) [file pcbi.1002407.s001.pdf]

Text S1: Supporting Information

Thermodynamic State Ensemble Models of *cis*-Regulation

Marc S. Sherman, Barak A. Cohen

Alternate *cis*-Regulatory Function Forms

Recall that the *cis*-regulatory functions used throughout the paper employ free concentrations of each biochemical species, and are thus referred to as "free species" form (Eq. 1). This equation is shown in the main text as Eq. 5.

$$< RNAP >= \frac{K_P[RNAP]}{1 + K_P[RNAP]} \tag{1}$$

Free Energy Form

Because K_P is a reflection of the Gibbs free energy of an interaction one may employ the fundamental relation between association constants and Gibbs free energy (Eq. 2) to rewrite Eq. 1 in terms of ΔG [1]. Solving for K_P and substituting the result into Eq. 1 yields Eq. 3.

$$\Delta G_P^\circ = -RTlnK_P \tag{2}$$

$$< RNAP >= \frac{e^{\frac{-\Delta G_P^\circ}{RT}}[RNAP]}{1 + e^{\frac{-\Delta G_P^\circ}{RT}}[RNAP]} \tag{3}$$

Written in this form, the dependence of K_P on temperature is laid bare, in turn highlighting the necessity of completing experiments under isothermal conditions. The relationship between temperature, Gibbs free energy, and enthalpy can also be leveraged to determine K_P using calorimetry and other *in vitro* methods [2,3].

Use of the association constant and corresponding free energy to describe binding of proteins to a single DNA site tacitly assumes that both are freely diffusing molecules at thermodynamic equilibrium. In actuality, the promoter we designate as "DNA" is a single rigid molecule in each cell, and transcription factors diffuse both freely and in one dimensional walks along DNA (see review, [4]). Ackers justifies the equilibrium assumption by arguing that the protein binding reactions occur on a much faster time scale than transcription and translation [1,5]. Thus, while the association constants are "apparent," and may not be the same as that measured in an *in vitro* system, they do have a physical interpretation and reflect important properties of the promoters being modeled.

State Form

It is often experimentally challenging to disentangle the contributions of the free species from their apparent association constants. In an experiment where one of these components is the independent variable–for example, if the sequence of a transcription factor binding site were modified–one may be able to discern individual components' contributions. In most other circumstances it is convenient to introduce a simplification where K_p and the concentrations of TFs are combined into a new variable q [6,7]. In our basal promoter example we have only two states, free DNA represented by the 1, and one additional state. Thus, we set $q_1 = K_P[RNAP]$ and Eq. 1 simplifies to Eq. 4, which contains only one unknown variable.

$$< RNAP >= \frac{q_1}{1 + q_1} \tag{4}$$

Fitting this model does not allow one to separate the effects of the binding constants from the effects of the concentrations of transcription factors. However this simplification of the model has fewer free parameters to fit and still allows for a physical interpretation of how a particular *cis*-regulatory system works.

Michaelis-like Functions with Basal Leak

To reconcile basal expression ambiguity, some groups [8–10] introduce a basal leak term, λ, to this formulation (Eq. 5) such that basal transcription occurs even in the absence of transcription factors.

$$\phi_m k_t = \lambda + (\prod_{i=1}^{m} A_i \prod_{j=1}^{n} R_j)c \tag{5}$$

A second new term, c, is also needed to scale the contribution of the transcriptional regulators, such that the maximum transcription rate, k_t, is given by Eq. 6 [8–10].

$$k_t = \lambda + c \tag{6}$$

As with the traditional Michaelis-like functions, the leak function can also be reconstituted as a thermodynamic model. Consider a basal promoter in the Michaelis-like leak formulation. In a basal promoter, transcription would not be influenced by an activator, for example ($c = 0$). Thus, production simplifies to λ [9]:

$$\phi_m k_t = \lambda \tag{7}$$

The production term for a basal promoter in the thermodynamic formulation is taken from Eq. 1.

$$\phi_m k_t = \frac{K_p[RNAP]}{1 + K_p[RNAP]} k_t \tag{8}$$

Then, setting these equal to each other,

$$\lambda = \frac{K_p[RNAP]}{1 + K_p[RNAP]} k_t \tag{9}$$

Substituting this definition of λ with Eq. 9 into a one activator model results in Eq. 10.

$$\phi_m k_t = \frac{K_p[RNAP]}{1 + K_p[RNAP]} k_t + \frac{\theta_A[A]}{1 + \theta_A[A]} c \tag{10}$$

Solving for c in Eq. 6 and substituting that into Eq. 10 results in Eq. 11 after simplification.

$$\phi_m = \frac{K_p[RNAP] + \theta_A[A] + K_p\theta_A[RNAP][A]}{1 + K_p[RNAP] + \theta_A[A] + K_p\theta_A[RNAP][A]} \tag{11}$$

Recalling that the numerator of a thermodynamic model is a sum of terms that encode states capable of transcribing while the denominator sums terms for all possible states, Eq. 11 shows that the leak model allows all states to transcribe except the state "1", where DNA is unbound (see Eq. 1). This includes the state where activator is bound and polymerase is not bound, represented by the $\theta_A[A]$ summand. When using a Michaelis-leak function one should be aware that this formulation implies that activators boost transcription even in the absence of RNA polymerase.

Oligomerization with Hill Functions

Hill-modified Michaelis-like functions (Eqs. 12 and 13 correspond to Eqs. 26 and 27 respectively in the main text):

$$A_i = \frac{(\theta_A[A])^n}{1 + (\theta_A[A])^n} \tag{12}$$

$$R_i = \frac{1}{1 + (\theta_R[R])^n} \tag{13}$$

A specific interpretation of the θ_A parameter allows Hill coefficients to encode oligomerization, an application of extreme cooperativity [9]. Using the thermodynamic model, we will demonstrate the relationship between integer-valued Hill functions and the corresponding oligomeric state of a DNA binding protein. Our example is for $n = 2$ (dimers) but holds for higher integer n values.

Consider a promoter with a single binding site for a dimer activator. Written in the same way as Eq. 1, the thermodynamic model for this situation is modeled in Eq. 14, where we make the Michaelis framework assumption that activator is absolutely required for transcription:

$$\phi_m = \frac{[A_2 \cdot RNAP \cdot DNA]}{[DNA] + [A_2 \cdot DNA] + [RNAP \cdot DNA] + [A_2 \cdot RNAP \cdot DNA]} \tag{14}$$

where A_2 represents an activator dimer. Dimer binds to free DNA with an association constant K_{A2},

$$K_{A2} = \frac{[A_2 \cdot DNA]}{[DNA][A_2]} \tag{15}$$

and dimer assembles from monomers with dimerization constant L_A:

$$L_A = \frac{[A_2]}{[A]^2} \tag{16}$$

Binding of polymerase and dimer is modeled with a macroscopic binding constant β_{AP}, which equals the right hand side of Eq. 17 in terms of stepwise equilibrium constants:

$$\beta_{AP} = \frac{[A_2 \cdot RNAP \cdot DNA]}{[A]^2[DNA][RNAP]} = K_P K_{A2} L_A \tag{17}$$

We showed previously how the $[RNAP \cdot DNA]$ term becomes $K_P[RNAP][DNA]$ (see Eq. 1). Making the appropriate substitutions into Eq. 14, we resolve the complete thermodynamic model for a dimer activator:

$$\phi_m = \frac{K_P K_{A2} L_A [A]^2 [RNAP]}{1 + K_P[RNAP] + K_{A2} L_A [A]^2 + K_P K_{A2} L_A [A]^2 [RNAP]} \quad (18)$$

The polymerase binding term can again be factored out.

$$\phi_m = \frac{K_{A2} L_A [A]^2}{1 + K_{A2} L_A [A]^2} \frac{K_P[RNAP]}{1 + K_P[RNAP]} \quad (19)$$

The right-most term is the basal promoter function and the term on the left is the activator function which we can again compare directly to Eq. 12. By setting the right-hand-side of Eq. 19 equal to Eq. 12, we see that $\theta_A^2 = K_{A2} L_A$, the product of the dimerization constant and the dimer-DNA association constant. Thus, while θ_A has no particular meaning, θ_A^2 is a macroscopic binding constant that describes the process of dimerization and DNA association. This exercise further emphasizes that the Hill/Michaelis-like functions are thermodynamic models that operate under several key assumptions.

References

1. Shea MA, Ackers GK (1985) The or control system of bacteriophage lambda. a physical-chemical model for gene regulation. J Mol Biol 181: 211–30.

2. Ye H, Wu H (2000) Thermodynamic characterization of the interaction between traf2 and tumor necrosis factor receptor peptides by isothermal titration calorimetry. Proc Natl Acad Sci USA 97: 8961–6.

3. Jacobson RH, Ladurner AG, King DS, Tjian R (2000) Structure and function of a human tafii250 double bromodomain module. Science 288: 1422–5.

4. Halford SE, Marko JF (2004) How do site-specific dna-binding proteins find their targets? Nucleic Acids Res 32: 3040–52.

5. Ackers GK, Johnson AD, Shea MA (1982) Quantitative model for gene regulation by lambda phage repressor. Proc Natl Acad Sci USA 79: 1129–33.

6. Buchler NE, Gerland U, Hwa T (2003) On schemes of combinatorial transcription logic. Proc Natl Acad Sci USA 100: 5136–41.

7. Gertz J, Siggia ED, Cohen BA (2009) Analysis of combinatorial cis-regulation in synthetic and genomic promoters. Nature 457: 215–8.

8. Mangan S, Alon U (2003) Structure and function of the feed-forward loop network motif. Proc Natl Acad Sci USA 100: 11980–5.

9. Sneppen K, Krishna S, Semsey S (2010) Simplified models of biological networks. Annu Rev Biophys 39: 43–59.

10. Alon U (2007) An introduction to systems biology: design principles of biological circuits. Boca Raton, FL: Chapman & Hall/CRC, 301 pp.
